# A Proactive Health Behavior Framework for Cognitive Impairment in Chinese Older Adults: Based on a Four-Factor and Logistic Regression Analysis

**DOI:** 10.3390/healthcare14020164

**Published:** 2026-01-08

**Authors:** Shengjiang Wang, Hailun Liang

**Affiliations:** 1 Department of Quality Management, Jinan Yifa Medical Laboratory, Jinan 250118, China; tolerance2002@163.com; 2 School of Public Administration and Policy, Renmin University of China, Beijing 100872, China

**Keywords:** cognitive dysfunction, proactive health behavior, AD-8 Scale, factor analysis

## Abstract

**Highlights:**

**What are the main findings?**

- Psychological–social support and information-behavior execution are major protective factors against screening positivity on the AD8 Dementia Screening Interview (AD8) among Chinese older adults; each one-standard-deviation increase reduces screening-positive risk by 39% and 53%, respectively;
- Age significantly increases cognitive impairment risk (21.7% per 5-year increment).

**What are the implications of the main findings?**

- Strengthening psychological support and optimizing health information access could be core strategies for dementia prevention;
- Integrating family and community resources with digital health technologies may enhance cognitive health equity.

**Abstract:**

**Objective**: In the context of an aging population, the prevention and control of cognitive impairment is a key public health priority. This study aims to investigate the association between proactive health behaviors and the risk of AD8 screening positivity in older adults in China, providing an empirical basis for developing targeted intervention strategies. **Methods**: Based on health behavior data from 1110 older adults in China, the chi-square test was used to analyze the differences in proactive health behaviors (such as limiting salt and alcohol intake, smoking cessation, and vaccination) between the low-risk and high-risk groups for AD8 screening. Factor analysis was used to extract the main factors of proactive health behaviors. Firth penalized logistic regression models were used to analyze the impact of the main factors and sociodemographic factors on the risk of cognitive impairment. **Results**: The chi-square test showed that there were significant differences between the two groups in salt restriction behavior (χ^2^ = 18.063, *p* < 0.01) and vaccination (χ^2^ = 29.674, *p* < 0.01), with a higher proportion of salt restriction (34.7%) and vaccination rates (80.4%) in the low-risk group. Factor analysis extracted four main factors (psychological–social support, information–behavior execution, technology–environment promotion, and addictive behavior control), with a cumulative variance contribution rate of 58.45%. Among them, psychological–social support (31.42% explained variance) and information–behavior execution (28.04%) had the strongest explanatory power. Firth penalized logistic regression showed that psychological–social support (Firth-corrected OR = 0.072, 95% CI: 0.035–0.148, *p* < 0.01) and information–behavior execution (Firth-corrected OR = 0.008, 95% CI: 0.003–0.021, *p* < 0.01) had significant protective effects on AD8 screening positivity (standardized OR values indicated that each one-standard-deviation increase in these two factors reduced screening-positive risk by 39% and 53%, respectively), and the risk increased by 21.7% for every 5-year increase in age (OR = 1.217, *p* = 0.001). Technology–environment promotion (OR = 0.417, 95% CI: 0.250–0.691, *p* = 0.001) and addictive behavior control (OR = 0.709, 95% CI: 0.490–1.026, *p* = 0.068) showed no significant protective effects. Sensitivity analysis confirmed the robustness of the four-factor structure and core conclusions. **Conclusions**: Among proactive health behaviors, psychological–social support and information–behavior execution are key protective factors in reducing the risk of AD8 screening positivity in older adults, and age is an important influencing factor. Strengthening psychological support and optimizing access to health information and behavior execution can serve as core strategies for cognitive impairment prevention and control, providing empirical support for the formulation of health policies for older adults.

## 1. Introduction

Cognitive dysfunction—spanning mild cognitive decline to severe dementia—reflects structural and/or functional brain abnormalities and constitutes an escalating challenge amid global population aging [1]. Although proactive health behaviors can attenuate cognitive deterioration [2,3], early identification of at-risk individuals is essential for targeted intervention. The AD8, as a screening tool for cognitive impairment, demonstrates good validity (with a sensitivity of 92% and a specificity of 96%) and facilitates rapid implementation among older adults [4]. In China, rapid demographic transition has produced a substantial and growing burden of cognitive impairment; however, a quantifiable association between proactive health behaviors and standardized AD8-defined outcomes has not been established in a nationally representative sample that covers both urban and rural residents. Moreover, existing studies on health behaviors and cognitive function mostly focus on single behaviors (such as exercise and diet), ignoring the comprehensive and multidimensional characteristics of proactive health behaviors. This study addresses this evidence gap by examining the theoretical framework of proactive health behaviors among AD8-screened older adults selected through a population-based sampling frame that mirrors the national age–sex–residence distribution, with the aim of informing precision dementia-prevention strategies across diverse Chinese settings.

## 2. Materials and Methods

### 2.1. Research Object

This data originates from the Psychological and Behavioral Investigation of Chinese Residents (PBICR-2021), employing quota sampling (the quota attributes being gender, age, and urban–rural distribution, conforming to the proportion of China’s ‘population pyramid’), totaling 1110 cases (576 cases in low-risk group and 534 cases in high-risk group), The high-risk group was determined by AD8 score ≥ 2 (screen-positive, requiring further clinical evaluation).

#### 2.1.1. Inclusion Criteria

(1) Age ≥ 60 years; (2) Chinese nationals; (3) Permanent resident (time spent away from home ≤ 1 month); (4) Signed informed consent form; (5) Able to actively complete questionnaire surveys; (6) Sufficient comprehension of all questionnaire items.

#### 2.1.2. Exclusion Criteria

(1) Individuals with impaired consciousness or mental abnormalities; (2) Individuals diagnosed with cognitive dysfunction; (3) Individuals with severe physical dysfunction (e.g., hearing impairment) who are unable to complete the questionnaire normally.

### 2.2. Research Instruments

#### 2.2.1. General Behavioral Data

(1) Smoking cessation behavior: Assign scores of 0, 1, and 2 to multiple instances of smoking in the past 12 months (not quit), smoking before 12 months but complete abstinence in the past 6 months (quit), and never smoking, respectively.

(2) Alcohol restriction behavior: No alcohol restriction refers to at least one instance of alcohol restriction behavior per week, moderate alcohol restriction refers to having drunk alcohol more than one time in the past 30 days with intervals exceeding 7 days, and complete alcohol restriction refers to no alcohol consumption in the past 12 months, assigned scores of 0, 1, and 2, respectively.

(3) Salt restriction behaviors: This includes reducing salt usage, using quantitative salt spoons, and selecting low-salt foods. Each behavior is scored as 1 point. For frequent consumption of pickled mustard tuber, pickles, and soy-fermented foods, 0 points are awarded. The total score is categorized into four levels: 0 (no salt restriction behavior), 1 (one type of salt restriction behavior), 2 (two types of salt restriction behaviors), and 3 (three or more types of salt restriction behaviors).

(4) Vaccination behavior: A score of 0 is assigned for non-vaccination with category II vaccines, and a score of 1 is assigned for vaccination.

(5) Smart home usage behavior: Visual Analog Scale Rating (0–100 points), assessing the frequency of use of smart devices (smart speakers/robots, etc.).

(6) Media usage behavior: Rate the frequency of using media such as newspapers, radio, computers, and smartphones. Assign scores of 0, 1, 2, 3, and 4 for never, occasionally, sometimes (2–3 days/week), often (4–5 days/week), and almost every day, respectively.

(7) Environmental health promotion behavior: Visual analog scale score (0–100 points), assessing support for low-carbon living and support for smoke-free environments.

#### 2.2.2. Analysis of Relevant Scales

A summary of the measurement instruments used to assess cognitive function and the dimensions of proactive health behaviors—including their purposes and internal consistency reliability (Cronbach’s α)—is provided in Table 1.

### 2.3. Statistical Methods

The statistical software IBM SPSS (Version 28.0; IBM Corp., Armonk, NY, USA) was used, and *p* < 0.05 indicated statistically significant differences. Categorical variables were described using frequency (n) and percentage (%), normally distributed numerical variables were described using mean ± standard deviation (x ± s), and skewed distributions were described using the median supplemented with the interquartile range [M (Q1,Q3)] for robust estimation. The former was analyzed using the chi-square test, and the latter was analyzed using the Mann–Whitney U test. The correlation between each proactive health behavior and the AD-8 scale score was analyzed using Kendall’s tau-b correlation analysis. Exploratory factor analysis was used for dimensionality reduction in the multidimensional analysis of proactive health behaviors. Prior to logistic regression analysis, collinearity diagnostics were performed using the Variance Inflation Factor (VIF) to evaluate potential multicollinearity among independent variables. To quantify their associations with AD8 screening positivity and to mitigate potential quasi-complete separation identified in preliminary analyses, we employed Firth penalized logistic regression as the primary analytical model. This method reduces parameter estimation bias by integrating a penalty term derived from Bayesian prior information. The Firth regression was implemented using the ‘logistf’ package (version 1.26.1; URL: https://CRAN.R-project.org/package=logistf, accessed on 18 February 2025) in R (version 4.4.3; R Foundation for Statistical Computing, Vienna, Austria. URL: https://www.R-project.org/), with default penalty parameters. The classification performance of the final model was evaluated using ROC curves. Finally, a sensitivity analysis was conducted by removing the salt restriction item and re-running the factor analysis and regression to verify the robustness of the core conclusions.

### 2.4. Ethical Review

Following all ethical principles, this study has passed ethical review (JKWH-2021-01).

## 3. Results

### 3.1. Descriptive Analysis of Proactive Health Behaviors

Chi-square tests of health behaviors among 1110 elderly Chinese individuals revealed significant differences in salt restriction (χ^2^ = 18.063, *p* < 0.01) and vaccination (χ^2^ = 29.674, *p* < 0.01) between the low-risk and high-risk groups of AD8 screening, while there were no differences in smoking cessation and alcohol restriction. The low-risk group had a significantly higher proportion of salt restriction measures (34.7% vs. 26.7%) and vaccination rates (80.4% vs. 65.7%); the proportion of unvaccinated individuals in the high-risk group was 1.8 times that of the low-risk group. Smoking and alcohol consumption were not significantly associated with the risk of AD8 screening positivity, which may be related to confounding factors (such as the positive correlation between smoking cessation/alcohol restriction and socioeconomic status) and limited variance in alcohol restriction behavior (72% of the sample were lifelong non-drinkers) (Table 2).

In addition to categorical health behaviors, significant disparities were observed in multiple proactive health behavior scales between the two risk groups (Table 3). Compared to the high-risk group, individuals in the low-risk group reported significantly higher scores for multidimensional exercise behavior, perceived social support, health maintenance, non-depressive and non-anxious psychological behaviors, smart product use, media use, and chronic disease self-management (* *p* < 0.05 for all). No significant intergroup differences were found in self-regulated diet, family mutual assistance, or environmental health promotion behaviors.

### 3.2. Results of Factor Analysis of Proactive Health Behavior

The results of the KMO test showed that the KMO value was 0.731, which is suitable for factor analysis; Bartlett’s test of sphericity showed a significant level (*p* < 0.05), indicating that factor analysis is effective. The number of principal factors was determined to be four based on the factor loading coefficient table and scree plot after orthogonal rotation with variance maximization.

Factor 1 (Psychological–Social Support): High-loading variables include psychological behavior-non-depression (0.830), non-anxiety (0.811), perceived social support (0.658), and healthy family mutual support behaviors (0.640), reflecting the positive effect of healthy family environment behaviors and social support on alleviating depression/anxiety. Factor 2 (Information–Behavior Execution): Media use (0.779), multidimensional exercise (0.698), and health maintenance (0.586) behaviors are dominant, revealing the association between information acquisition and health behavior execution, emphasizing the critical role of media in health literacy. Factor 3 (Technology–Environment Promotion): Smart home use (0.814) and environmental health promotion behaviors (0.728) are central, reflecting the dual drive of technology application and low-carbon, smoke-free environments on health behaviors. Factor 4 (Addictive Behavior Control): Smoking cessation (0.821) and alcohol restriction (0.818) are highly clustered, representing the individual’s proactive avoidance of health risks. The communality shows that the explanatory power of most variables is >0.4, but salt restriction behavior (0.258) and vaccination (0.275) are affected by multiple factors (such as regional dietary cultural specificity, individual taste preferences, and medical resource accessibility), requiring further verification through sensitivity analysis.

The weight calculation results show that four principal factors were extracted, with a cumulative variance contribution rate of 58.45%. Factor 1 (psychological–social support, 31.42%) has the strongest predictive power, followed by factor 2 (behavioral execution–information acquisition, 28.04%), with a cumulative explanation rate of 34.75%; factor 3 (technology–environment interaction, 21.00%) and factor 4 (addiction control, 19.55%) have weaker predictive power and similar weights (Table 4 and Table 5).

### 3.3. Firth Penalized Logistic Regression Analysis of Proactive Health Behavior Factors

The logistic regression model (forward stepwise method), with a likelihood ratio chi-square value of 1372 (*p* < 0.01), Hosmer–Lemeshow test (*p* = 0.664), and Nagelkerke R^2^ = 0.236, demonstrates good model fit. Among sociodemographic factors, only age is significant (OR = 1.217, *p* = 0.001), with the risk increasing by 21.7% for every 5-year increase. Factor 1 (Psychological–Social Support) and Factor 2 (Information–Behavior Execution) exhibit the strongest protective effects (Firth-corrected OR = 0.072, 95% CI: 0.035–0.148; OR = 0.008, 95% CI: 0.003–0.021, both *p* < 0.01), standardized OR values indicated that each one-standard-deviation increase in these two factors reduced screening-positive risk by 39% and 53%, respectively; Factor 3 (Technology–Environment) reduces risk by 58.45% (OR = 0.417, 95% CI: 0.250–0.691, *p* = 0.001); Factor 4 (Addiction Control) did not show significant protective effects (OR = 0.709, 95% CI: 0.490–1.026, *p* = 0.068). The model’s performance was evaluated using ROC curve analysis, demonstrating good performance (accuracy of 0.674, AUC value of 0.731) (Table 6).

## 4. Discussion

### 4.1. Research Status and Significance

With the aggravation of population aging, the problem of cognitive impairment in older adults is becoming more and more serious, which seriously affects the quality of life of older adults and brings a huge burden to the family and society. According to the World Alzheimer’s Disease Report 2023 [13], it is estimated that by 2050, the number of patients with dementia worldwide will increase from 55 million in 2019 to 150 million. At present, the number of dementia patients in China has exceeded 10 million, and it is expected to exceed 40 million by 2050. The Healthy China Action Plan (2019–2030) has listed “the decline in the growth rate of Alzheimer’s disease among people aged 65 and over” as one of the key objectives [14].

Existing studies have shown that proactive health behaviors are the key intervention measure to delay cognitive impairment. Proactive health behaviors refer to the positive actions taken by individuals in order to maintain and promote their own health, including reasonable diet, moderate exercise, smoking cessation and alcohol restriction, active social interaction, psychological adjustment, and health information acquisition and utilization. According to the residents’ mastery of health-related knowledge and the implementation of health-related behaviors, foreign scholars divide proactive health behaviors into two parts: health literacy and health behavior [15]. Health literacy refers to the ability of individuals to obtain and understand basic health information and services, and use these information and services to make correct health decisions [16]. The purpose of this study is to build a theoretical framework of proactive health behaviors in the prevention of cognitive dysfunction by analyzing the correlation between proactive health behaviors and AD8 screening positivity of older adults in China, so as to provide the basis for formulating effective prevention and control strategies, so as to improve the cognitive health status of older adults and reduce the social burden, which has important social value.

### 4.2. Proactive Health Behavior Intervention Theory

#### 4.2.1. Four Factors and Their Theoretical Mechanisms

Construct a four-dimensional framework of “psychological–social support, information acquisition–behavior execution, technology–environment promotion, and addiction behavior control”, and combine the three-level amplification mechanism of “individual ability (health literacy), family function (health mutual assistance), and community resources (supportive promotion)”. Based on factor analysis and Firth penalized logistic regression, the intervention priority model of “psychosocial support > information empowerment > scientific and technological adaptation” was established to reveal the dynamic balance mechanism of “psychology behavior environment”, and provide a theoretical basis for hierarchical intervention.

Psychosocial–Social support: social support and family mutual aid enhance psychological resilience through emotional connection and resource supply. Its high-load characteristics confirm the “micro system” theory of human development ecology and reveal the synergistic gain effect of mental health and social support. The pressure buffering theory verified that social support affected health behavior choice by regulating psychological stress, and the weight of factor 1 was 1.89 times that of factor 4, suggesting that the traditional intervention strategy focusing on smoking cessation and alcohol restriction may have the risk of health resource mismatch. Information–behavior execution: Based on the theory of health action process orientation (HAPA), media use drives multidimensional sports behavior by acquiring digital health knowledge. Health literacy, as the cognitive basis, cooperatively promotes the transformation of “plan action” by improving information screening efficiency and enhancing self-efficacy. WHO data confirmed that there was a dose effect between health literacy and exercise compliance (or = 1.12) [17]. The “behavior amplifier” effect of factor 2 (or =0.007) reflected the key role of information transformation ability in the sustainability of health behavior. Technology–environment promotion: Based on health ecology, the smart home factor breaks through the space–time limit by adjusting the individual environment matching degree through the IEM index. However, this factor did not show significant protective effects on AD8 screening positivity in this study, which may be due to the low adoption rate of smart devices in the older adult population (average VAS score < 60 points) and the lagging effect of environmental interventions on cognitive health. Control of addictive behavior: independent high load (>0.8) confirms that substance dependence management follows the self-regulation theory and is essentially different from conventional health behavior. The lack of significant association with AD8 screening positivity may be due to the cumulative damage effect of smoking/drinking—long-term exposure increases cognitive risk with a time lag [18,19], which cannot be fully captured by cross-sectional data. It is necessary to analyze the nonlinear trajectory in the theory of behavior transition stages through the potential growth model to reveal the separation of addiction withdrawal and the health promotion behavior mechanism.

Individual health literacy is the foundation that drives the chain of knowledge from the acquisition of tools to the application and maintenance of behaviors, and healthy psychology enhances individual behavior persistence. Family function is the core link. Family members assist in smoking cessation, alcohol and salt restriction, and healthy diet and exercise, and alleviate poor psychology through family interaction and support. At the community level, resource integration is the fulcrum of leverage. The community provides fitness venues to promote multidimensional sports. Through health-themed activities, individuals can understand social support, promote self-management, and change the pattern of group behavior.

#### 4.2.2. Interpretation of Non-Significant Findings

Smoking cessation and alcohol restriction: The absence of significant differences between groups may be attributed to three factors: (1) Confounding interference: Data from this study show that smoking cessation and alcohol restriction are significantly positively correlated with education level and household income (r = 0.182, r = 0.157, both *p* < 0.01), and their independent effects are diluted after adjusting for sociodemographic and core behavioral factors. (2) Limited variance: The proportion of lifelong non-drinkers in the sample reaches 72%, resulting in insufficient variability in alcohol restriction behavior and reduced statistical power. (3) Incomplete measurement: Only the “status” (yes/no) of smoking cessation and alcohol restriction was collected, without capturing key information such as abstinence duration and alcohol dosage. The relevant literature, including a dose–response meta-analysis [18], suggests that cognitive benefits exhibit a significant dose–response relationship [19]. Therefore, this result cannot be interpreted as smoking cessation and alcohol restriction having no effect on cognitive health, but only indicates that their independent effects are not prominent in the current behavioral framework.

Technology–environment promotion: The non-significant protective effect may be related to the low adoption rate of smart devices in the older adult population (less than 15% of the sample used smart health tools frequently) and the lagging nature of environmental interventions—changes in home environment and technology use may require a longer follow-up period to show cognitive benefits. This suggests that in current cognitive risk interventions for community-dwelling older adults, priority should be given to evidence-based strategies such as psychological–social support and health information execution, while technology–environment interventions can be regarded as a long-term potential direction.

### 4.3. Risk Prevention and Control Strategies of Cognitive Impairment Based on Proactive Health Behavior Theory

Older adults should pay attention to mental health, improve health literacy, perform well in health self-management, scientific diet and exercise management, and promote personal health management through science and technology. The mutual assistance of family members’ health action and mutual supervision of healthy diet and exercise should be strengthened, and the control of smoking cessation, alcohol and salt restriction and other unhealthy behaviors should be promoted. The community should strengthen mental health services, carry out social care activities, promote the development of sports, and create an intelligent aging, low-carbon, and smoke-free community environment. Policy makers should optimize the allocation of resources, formulate policies reasonably, promote the participation of the whole society, and guide enterprises to use modern technology to develop elderly care intelligent products that adapt to the characteristics of different groups of people.

### 4.4. Study Limitations

This study has several limitations that should be considered when interpreting the findings and addressed in future research. First, the cross-sectional design, based on PBICR-2021 data, can only identify associations between proactive health behaviors and AD8 screening positivity; it cannot establish causality. The temporal sequence between variables (e.g., whether proactive behaviors precede cognitive changes) remains unclear, and reverse causality (e.g., early cognitive decline influencing health behaviors) cannot be ruled out.

Second, most proactive health behaviors (e.g., smoking cessation, alcohol restriction) were assessed via self-report, which is subject to recall bias, social desirability bias, and subjective interpretation. Furthermore, the outcome was defined by AD8 screening positivity—a screening tool, not a clinical diagnosis. Thus, the results reflect associations with screening outcomes, not clinically diagnosed cognitive impairment.

Third, the precision of behavioral measurement was limited. Key behaviors were captured as categorical variables without details on duration or intensity, potentially affecting effect estimates. Additionally, the variable for salt restriction loaded weakly (communality = 0.258) within the four-factor framework, possibly due to its strong ties to regional dietary culture and taste preferences. While sensitivity analyses confirmed the robustness of the main findings, the factor structure warrants validation in more diverse samples.

Fourth, statistical adjustments were required due to data characteristics. Preliminary analysis indicated quasi-complete separation, evidenced by large standard errors and wide confidence intervals. We therefore applied Firth penalized logistic regression to stabilize parameter estimates. While effective, this approach addresses a data limitation that ideally would be avoided through more precise sampling and measurement in future designs.

Fifth, residual confounding may persist. Although age and education were adjusted for, other potential confounders—such as socioeconomic status, comorbid chronic conditions, genetic factors (e.g., APOE ε4), and lifestyle variables not captured by the four-factor framework—were not measured and could influence the observed associations.

Finally, the sample’s regional representativeness may be limited. Although quota sampling was used to match national age–sex–residence distributions, regional variations in dietary culture (e.g., salt intake) and healthcare access may affect the generalizability of results. Future studies should include broader geographic coverage to enhance external validity.

## 5. Conclusions

This study establishes a proactive health behavior framework, identifying psychosocial support and information–behavior execution as key protective factors against cognitive impairment in older adults. These findings advocate for integrated interventions that strengthen psychological resilience, family support, and health literacy. Future longitudinal studies are needed to confirm the causal pathways and elucidate the underlying mechanisms.

## Figures and Tables

**Table 1:** Overview of scales related to proactive health behaviors.

Health Behavior	Assessment Scale	Purpose Description	Cronbach’s α
	Dementia Screening Scale AD8	Used for screening cognitive impairment; simple and rapid identification of at-risk populations [5,6,7]	0.930
Rational Diet Behavior	Eating Behavior Scale (EBS-SF)	Evaluate the self-regulated dietary behavior of Participants [8]	0.880
Multidimensional Exercise Behavior	National Fitness Campaign NFC	Assess the frequency, intensity, duration, and type of physical activity participated in by Participants	0.801
Healthy Family Mutual Aid	Healthy Family Scale FHS-SF	Evaluate family health mutual aid and collaborative health management effectiveness [9]	0.940
Perceived Social Support	Perceived Social Support Scale (PSSS)	Assess the overall level of social support perceived by Participants	0.952
Health Maintenance Behavior	Health Literacy Scale HLS-SF12	Evaluate medical resource utilization, disease control, and health promotion among Asian populations [10]	0.921
Psychological Behavior Non-Depression	Patient Health Questionnaire-9 (PHQ-9)	Assess depressive psychological behaviors of Participants including emotions, sleep, diet, and attention [11]	0.922
Psychological Behavior Non-Anxiety	Generalized Anxiety Disorder 7-Item Scale GAD-7	Assess generalized anxiety psychological behaviors of Participants over the past two weeks [12]	0.946
Chronic Disease Management Behavior	Chronic Disease Self-Management Scale CDSMS	Evaluate the self-efficacy and adaptability of chronic disease patients in disease management	0.945

**Table 2:** Health behaviors of elderly patients for AD8 screening (qualitative data) (*n* = 1110).

Variable	Options	Population Classification *n* (%)		χ^2^	*p*
		Low-Risk Group	High-Risk Group		
Smoking Cessation Behavior	Not Quitted	74 (12.8)	84 (15.7)	1.890	0.389
	Quitted	119 (20.6)	106 (19.8)		
	Never Smoked	383 (66.5)	344 (64.4)		
Alcohol Restriction Behavior	No Restriction	102 (17.7)	82 (15.3)	1.116	0.572
	Moderate Restriction	87 (15.1)	82 (15.3)		
	Complete Restriction	387 (67.2)	370 (69.3)		
Salt Restriction Behavior	None	69 (11.9)	106 (19.8)	18.063	0.001 *
	One Type	89 (15.4)	96 (17.9)		
	Two Types	218 (37.8)	189 (35.4)		
	Three or More	200 (34.7)	143 (26.7)		
Vaccination Behavior	Not Vaccinated	113 (19.6)	183 (34.3)	29.674	0.080
	Vaccinated	463 (80.4)	351 (65.7)		

Note: Data are presented as mean ± SD or *n* (%). * *p* < 0.05.

**Table 3:** Health behavior scale for elderly patients for AD8 screening (quantitative data) (*n* = 1110).

Variable	Population Classification (M [Q1, Q3])		Mann–Whitney U Statistic	*p*
	Low-Risk Group	High-Risk Group		
Self-Regulated Diet Behavior	21 [17, 25]	21 [18, 24]	145,577	0.122
Multidimensional Exercise Behavior	4 [3, 8]	3 [2, 4.75]	191,895	0.000 *
Family Mutual Assistance Behavior	20 [17, 24]	20 [18, 23]	153,959	0.975
Perceived Social Support Behavior	61 [51, 72]	48 [39.25, 57]	242,268	0.000 *
Health Maintenance Behavior	36 [32, 37]	32 [28, 36]	206,533	0.000 *
Non-Depressive Psychological Behavior	25 [19, 27]	22 [18, 25]	193,064	0.000 *
Non-Anxious Psychological Behavior	20 [15, 21]	17 [14, 21]	187,530	0.000 *
Smart Product Use Behavior	60 [28.75, 83]	56 [23, 80]	164,741	0.040 *
Media Use Behavior	12 [7, 15]	8 [4, 13]	191,602	0.000 *
Environmental Health Promotion Behavior	79 [58, 98.5]	79 [56.62, 99]	155,107	0.804
Chronic Disease Self-Management Behavior	11 [6, 15]	10 [6, 13]	170,667	0.002 *

Note: Data are presented as mean ± SD or *n* (%). * *p* < 0.05.

**Table 4:** Factor analysis of proactive health behavior factors—table of factor loading coefficients.

Variable	Rotated Factor Loading Coefficients				Communality
	Factor 1	Factor 2	Factor 3	Factor 4	
Smoking Cessation Behavior	0.027	−0.015	0.019	0.821	0.676
Alcohol Restriction Behavior	−0.04	−0.108	0.022	0.818	0.684
Salt Restriction Behavior	0.126	0.259	0.314	0.278	0.258
Self-Regulated Diet Behavior	0.576	−0.206	0.008	0.108	0.386
Multidimensional Exercise Behavior	−0.194	0.698	0.083	−0.065	0.535
Family Mutual Assistance Behavior	0.640	0.149	0.070	0.099	0.446
Perceived Social Support Behavior	0.658	0.339	0.174	0.083	0.586
Health Maintenance Behavior	0.404	0.586	0.252	0.043	0.572
Non-Depressive Psychological Behavior	0.830	−0.103	0.025	−0.105	0.711
Non-Anxious Psychological Behavior	0.811	−0.059	−0.023	−0.122	0.677
Smart Home Device Use Behavior	−0.032	0.014	0.814	−0.121	0.679
Media Use Behavior	−0.031	0.779	−0.036	−0.047	0.611
Environmental Health Promotion Behavior	0.210	0.041	0.728	0.166	0.604
Vaccination Behavior	0.072	0.519	0.013	−0.009	0.275
Chronic Disease Self-Management Behavior	−0.302	0.419	0.383	0.002	0.413

Note: Salt restriction behavior is a four-level categorical indicator with a factor communality of 0.258. The remaining variance (74.2%) may be attributed to unique factors such as the high-salt dietary tradition in the study area.

**Table 5:** Factor analysis of proactive health behaviors—factor weight analysis.

Variable	Variance Explained (%)	Cumulative Variance Explained (%)	Weight (%)
Factor 1	18.36	18.36	31.42
Factor 2	16.39	34.75	28.04
Factor 3	12.27	47.02	21
Factor 4	11.43	58.45	19.55

**Table 6:** Results of Firth penalized logistic regression analysis of proactive health behavior factors.

Variable	β	SE	Wald χ^2^	*p*	OR	OR (95% CI)		Standardized OR (per1SD)
						Lower	Upper	(95% CI)
Factor 1	−2.628	0.368	50.126	0.000	0.072	0.035	0.148	0.612 (0.381–0.984)
Factor 2	−4.898	0.538	83.742	0.000	0.008	0.003	0.021	0.466 (0.272–0.797)
Factor 3	−0.875	0.259	11.432	0.001	0.417	0.250	0.691	0.789 (0.576–1.080)
Factor 4	−0.344	0.188	3.328	0.068	0.709	0.490	1.026	0.889 (0.663–1.191)
Age	0.196	0.061	10.216	0.001	1.217	1.079	1.372	-
Educational Attainment	−0.075	0.075	1.006	0.316	0.928	0.801	1.074	-
Constant	3.988	0.504	62.153	0.000	53.943	20.304	146.959	-

Note 1: Standardized OR reflects the change in AD8 screening-positive risk per one-standard-deviation increase in the factor score; Factor 3 and Factor 4 showed no significant protective effects after comprehensive evaluation. Note 2: All variance inflation factors (VIFs) were low (Factor 1: 1.36, Factor 2: 1.06, Factor 3: 1.28, Factor 4: 1.37, Age: 1.38, Educational attainment: 1.08), confirming the absence of significant multicollinearity.

## Data Availability

The datasets generated and analyzed during the current study are available from the corresponding author upon reasonable request due to privacy restrictions.
